# Altered mitochondrial functionality in metabolic disorders: insights from *Drosophila* studies

**DOI:** 10.3389/fendo.2026.1817121

**Published:** 2026-05-19

**Authors:** Yunlin Zeng, Yang Yang, Yuao Zhu, Peixin Xu, Chakin Cheong, Yong Juan Zhao, Gyeong Hun Baeg

**Affiliations:** 1 Faculty of Health Sciences, University of Macau, Taipa, Macao SAR, China; 2 Faculty of Medicine, Macau University of Science and Technology, Taipa, Macao SAR, China; 3 Ciechanover Institute of Precision and Regenerative Medicine, School of Medicine, The Chinese University of Hong Kong, Shenzhen, China

**Keywords:** *Drosophila melanogaster*, mitochondrial dysfunction, mitochondrial quality control, mitophagy, obesity and type 2 diabetes

## Abstract

The pathophysiological association between obesity and type 2 diabetes (T2D) increasingly highlights the central role of mitochondrial dysfunction. As critical signaling hubs orchestrating metabolism, mitochondria are pivotal in maintaining metabolic homeostasis. Imbalances in mitochondrial quality control mechanisms lead to an accumulation of damaged mitochondria with abnormal dynamics and functions, exacerbating the progression of obesity, insulin resistance, and T2D. Although therapeutic interventions for obesity and T2D have shown promise, they remain insufficient for achieving sustained remission from obesity and T2D on a global scale. Furthermore, existing rodent models often struggle to fully recapitulate human metabolic disorders due to species-specific metabolic differences and technical limitations. *Drosophila melanogaster* has emerged as a powerful model organism for deciphering mitochondrial-metabolism interactions due to numerous advantages, including easy genetic manipulation, low gene redundancy, rapid phenotype verification, and the unique opportunity to image live tissues *in vivo*. *Drosophila* models effectively recapitulate high-sugar- and high-fat-diet-induced mitochondrial fragmentation, adipose tissue expansion, and insulin resistance-like phenotypes. Furthermore, studies leveraging the genetic tractability of *Drosophila* have provided critical insights into how mitochondrial impairment contributes to systemic metabolic dysfunction. Here, we introduce recent advances in mitochondrial research regarding metabolic disorders and demonstrate how *Drosophila* serves as a useful *in vivo* model to dissect mitochondrial function. Future research should integrate multi-omics approaches and precision medicine strategies targeting mitochondrial metabolic remodeling to break the vicious cycle of obesity and T2D, while developing non-invasive intervention methods to advance translational medicine.

## 1 Introduction

Obesity and type 2 diabetes (T2D) represent two interconnected global health crises. Obesity is clinically defined as a chronic condition characterized by excessive adiposity, leading to organ dysfunction or increased risks of metabolic disorders (1). Diabetes, characterized by insulin resistance and hyperglycemia, affected an estimated 828 million adults globally (~14% of adults) in 2022, with projections indicating a 46% increase in prevalence by 2045. Because more than 90% of diabetes cases are type 2, this burden largely reflects T2D (2–4). The surge in T2D parallels the global rise in obesity driven by sedentary lifestyles, high-calorie diets, and socioeconomic disparities (5, 6). Recent estimates suggest that 31.5% of adults in high-income nations will be obese by 2030 (7), and the prevalence of overweight among children and adolescents aged 5–19 years has risen dramatically from 8% in 1990 to 20% in 2022 (8), further exacerbating the burden of metabolic disorders. The pathophysiological interplay between obesity and T2D is rooted in shared mechanisms such as insulin resistance, chronic inflammation, and ectopic lipid deposition. Adipose tissue dysfunction promotes glycolysis and free fatty acid (FFA) release, impairing insulin signaling in skeletal muscle and the liver, thereby contributing to a vicious cycle of hyperglycemia and dyslipidemia (9). Hyperglycemia worsens insulin resistance and increases FFA release, driving dyslipidemia, whereas dyslipidemia further impairs insulin sensitivity, and aggravates hyperglycemia (10). Prospective cohort studies confirm a significantly elevated risk of T2D in individuals with overweight or obesity compared with normal-weight counterparts, while glucotoxicity and pancreatic β-cell dysfunction further complicate weight management in T2D patients (11). This bidirectional relationship underscores the need for integrated therapeutic strategies targeting both conditions to mitigate their combined morbidity and mortality.

**Figure 1:**
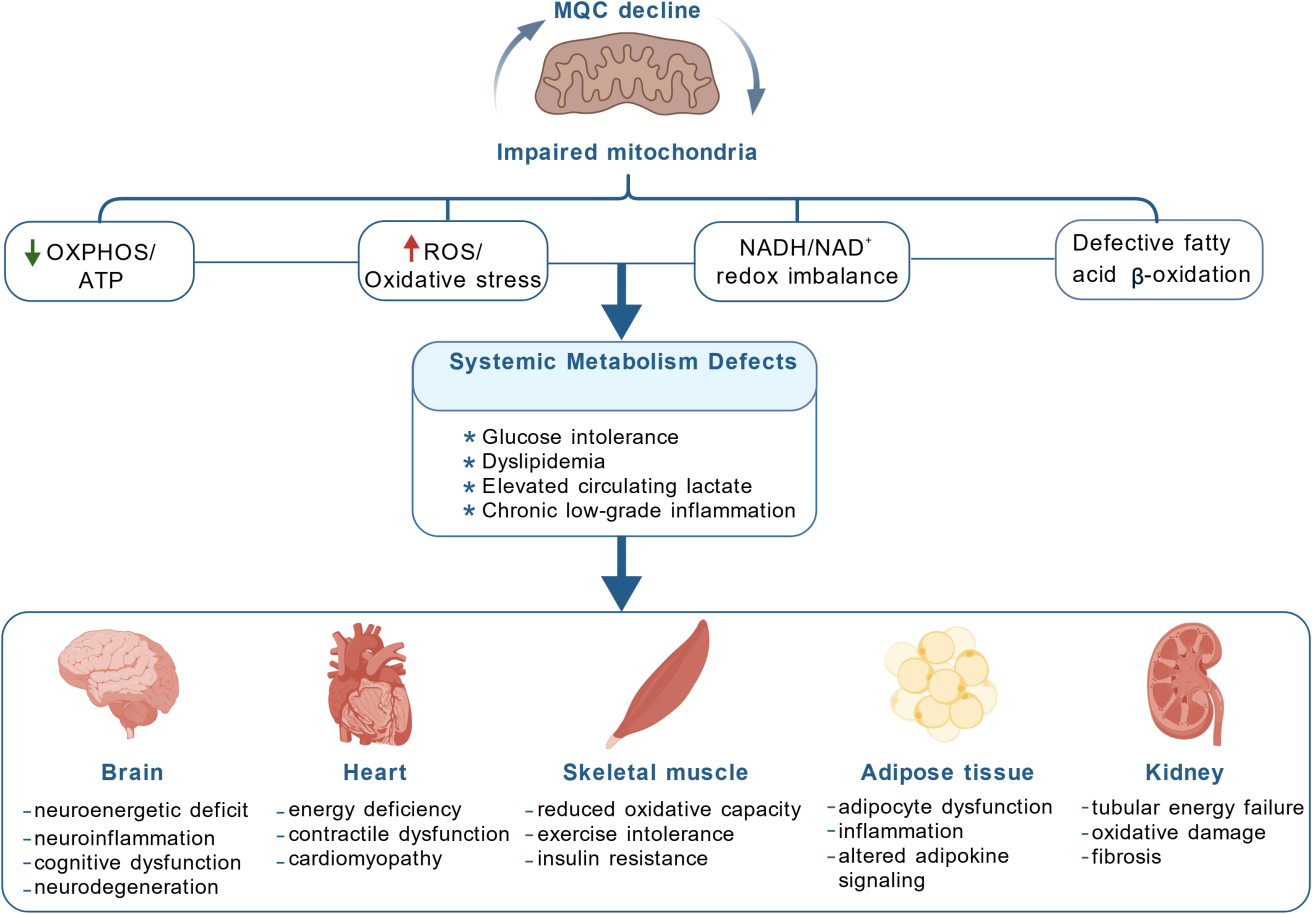
Mitochondrial dysfunction contributes to the pathogenesis of metabolic disorders. Mitochondrial dysfunction induced by a decline in mitochondrial quality control (MQC) leads to the accumulation of damaged mitochondria, causing reduced OXPHOS and ATP production, increased mitochondrial reactive oxygen species (ROS) and oxidative stress, disrupted NADH/NAD^+^ redox homeostasis, and impaired fatty acid β-oxidation. These abnormalities trigger systemic metabolic defects, leading to various disorders at the organ level.

Mitochondria are metabolic hubs that integrate energy homeostasis rather than merely serving as ATP-producing organelles (12–15). In metabolically active tissues such as the liver, adipose tissue, and skeletal muscle, mitochondrial metabolism not only determines substrate utilization and the efficiency of energy conversion, but also finely regulates cell fate and stress responses through the production of reactive oxygen species (ROS), tricarboxylic acid (TCA) cycle intermediates, and various metabolite-derived signaling molecules (13, 16, 17). For instance, in immune cells, mitochondrial metabolites and mitochondrial DNA (mtDNA) directly regulate pattern recognition receptor signaling, inflammasome activation, and inflammatory cytokine secretion, thereby establishing a connection between energy status and inflammation (18). Hence, mitochondria rely on a series of highly coordinated quality control mechanisms to maintain their dual roles in metabolism and immunity. Mitochondrial dysfunction contributes to the pathogenesis of various metabolic disorders (**Figure 1**). Mitochondrial dysfunction often first manifests as an imbalance in the mitochondrial quality control (MQC) network under various pathological stimuli. The ability of mitophagy to remove damaged mitochondria declines, mitochondrial biogenesis fails to compensate adequately, and mitochondrial dynamics (fusion/fission) and protein homeostasis are disrupted, leading to an accumulation of dysfunctional mitochondria and the continuous amplification of downstream pathological signals (19–23). As dysfunctional mitochondria accumulate and the efficiency of the respiratory chain decreases, oxidative phosphorylation (OXPHOS) is impaired and ATP supply becomes insufficient (24). ROS production also increases, accompanied by enhanced oxidative stress and disruption of NADH/NAD^+^ redox homeostasis. These changes promote cellular metabolic reprogramming, which can manifest as enhanced glycolysis and increased lactate production in various cell types (25). Collectively, these cellular-level bioenergetic and substrate utilization abnormalities translate into organ-specific functional consequences, such as neural energy disorders in the brain, decreased contraction or movement ability in cardiac and skeletal muscle, insulin resistance in adipose tissue, and fibrosis-associated energy insufficiency in proximal renal tubules.

Mitophagy acts as a crucial “safety valve” that links MQC, energy homeostasis, and inflammation control by eliminating defective mitochondria to maintain respiratory chain function, limit excessive mtROS production, and prevent abnormal leakage of mtDNA, thereby avoiding misactivation of cytoplasmic DNA sensors and inflammasomes (26–28). The accumulation of damaged mitochondria promotes chronic inflammation and energy metabolism disorders (29). Meanwhile, mitochondrial dynamics determine mitochondrial functional status by reshaping the morphology and network structure of mitochondria. Fusion mediated by Mitofusin 1/2 (MFN1/2) and Optic atrophy 1 (Opa1) facilitates the mixing of mitochondrial contents and enhances the efficiency of the respiratory chain, while fission led by Dynamin-related protein 1 (Drp1) isolates and eliminates damaged mitochondria (30, 31). In parallel, mitochondrial biogenesis, orchestrated by the transcriptional coactivator peroxisome proliferator-activated receptor-gamma coactivator 1-alpha (PGC-1α) and its downstream nuclear partners, adjusts mitochondrial mass, respiratory capacity, and antioxidant defenses in response to nutrient status, hormones, and inflammatory factors (32–34).

Overall, these coordinated processes shape mitochondria as a platform integrating energy metabolism, organelle turnover, and immune signaling. A thorough understanding of these regulatory processes is essential for elucidating how mitochondrial dysfunction contributes to metabolic disorders and for guiding the development of more effective targeted intervention strategies. In this context, *Drosophila* melanogaster provides a valuable *in vivo* model for studying metabolic disorders associated with mitochondrial dysfunction. Although simpler than mammalian systems, *Drosophila* preserves many evolutionarily conserved pathways involved in insulin signaling, lipid and glucose metabolism, mitochondrial quality control, and stress responses, while offering unique advantages such as low gene redundancy, rapid life cycle, powerful genetic tools, and tissue-specific manipulation. These features make *Drosophila* models particularly useful for dissecting how mitochondrial dysfunction drives systemic metabolic imbalance and organ-specific pathology. In this review, we first summarize the role of mitochondrial dysfunction in obesity and T2D and the limitations of current therapeutic and mammalian model systems and then discuss how *Drosophila* models have advanced our understanding of mitochondrial quality control, mitochondrial dynamics, mitophagy, and related metabolic phenotypes, as well as the experimental toolkit available for studying these processes *in vivo*. mammalian model systems and then.

## 2 Mitochondrial dysfunctions in obesity and diabetes: key mechanisms and conserved pathways

**Figure 2:**
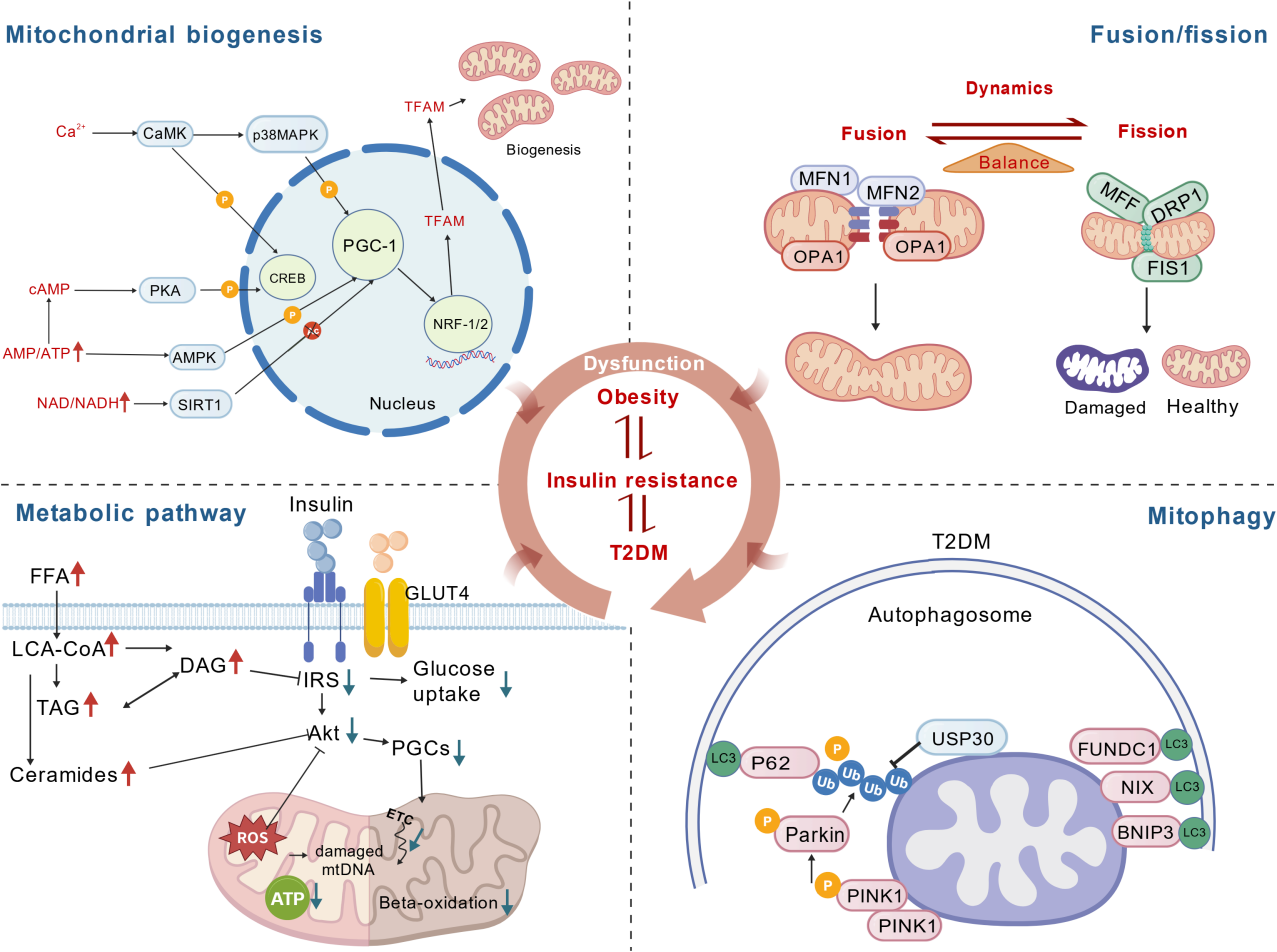
Mitochondrial pathways and their dysregulation in obesity and T2D. The transcriptional co-activator network centered on PGC-1α integrates upstream signals such as CaMK/p38 MAPK, cAMP/PKA, and AMPK/SIRT1 to activate NRF-1/NRF-2 and TFAM, thereby promoting mitochondrial biogenesis. Mitochondrial fusion is mediated by MFN1/2 and OPA1, and mitochondrial fission is mediated by MFF, DRP1, and FIS1. These molecules act to maintain the dynamic equilibrium of the mitochondrial network and enable the segregation of healthy and damaged mitochondria. Lipid intermediates such as FFAs, DAG/TAG, and ceramides impair IRS-1/PI3K/Akt signaling and GLUT4 translocation, while increased mitochondrial ROS, insufficient ATP production, and impaired fatty-acid β-oxidation contribute to the development of insulin resistance. The PINK1-Parkin pathway, together with LC3, p62, VDAC, FUNDC1 and NIX/BNIP3, and the deubiquitinating enzyme USP30, collectively regulate the selective autophagic clearance of damaged mitochondria. Dysregulation of mitochondrial biogenesis, dynamics, mitophagy, bioenergetics, redox balance, and lipid signaling ultimately contributes to obesity, insulin resistance, and the onset and progression of T2D.

Mitochondrial dysfunction in obesity and T2D is multifactorial and involves abnormalities in mitochondrial biogenesis, dynamics, mitophagy, bioenergetics, redox homeostasis, and lipid signaling, all of which contribute to insulin resistance and disease progression (**Figure 2**).

### 2.1 Mitochondrial biogenesis and mitohormesis

Mitochondrial biogenesis is coordinated by the PGC-1 family of co-activators, which integrate diverse nutritional, hormonal, and inflammatory signals to regulate the expression of nuclear and mitochondrial genes, thereby controlling OXPHOS, fatty acid oxidation, and antioxidant defense. Moderate mitochondrial stress can induce mitohormesis (35, 36), in which mild stressors such as exercise, moderate increases in ROS, or transient nutrient stress activate mitochondrial biogenesis through the Sirtuin 1(SIRT1)/AMP-activated protein kinase(AMPK)–PGC-1 axis (37–39), thereby enhancing antioxidant capacity and energy expenditure and conferring protection against obesity and T2D (38, 40, 41). Transcriptomic and population genetics studies have shown that, in skeletal muscle and adipose tissue of obese and T2D patients, genes involved in oxidative metabolism through the PGC-1α/nuclear respiratory factor 1 (NRF1)/mitochondrial transcription factor A (TFAM) axis are coordinately downregulated, which is associated with reduced mitochondrial content and impaired oxidative capacity, suggesting that defective mitochondrial biogenesis contributes to insulin resistance (42).

Beyond regulating mitochondrial abundance, mitochondrial biogenesis and mitohormesis are dynamically linked to mitochondrial fusion/fission balance, ROS homeostasis, and insulin sensitivity. Under physiological conditions, mild mitochondrial stress and moderate ROS production can activate adaptive mitohormetic responses, promote PGC-1α-dependent mitochondrial biogenesis, and support a more functionally integrated mitochondrial network (35). In contrast, under chronic nutrient excess and metabolic stress, as in obesity and T2D, ROS production becomes excessive and persistent, contributing to redox imbalance, impaired insulin signaling, and a shift toward excessive mitochondrial fragmentation (30, 43). Although compensatory mitochondrial biogenesis may be triggered under these conditions, it is frequently inadequate to fully restore mitochondrial network integrity and oxidative capacity. By contrast, exercise-associated mild ROS elevation promotes adaptive mitochondrial remodeling, including enhanced biogenesis and a shift toward a more fused mitochondrial network, thereby improving oxidative metabolism and insulin sensitivity (35, 44). Collectively, these findings indicate that the metabolic outcome depends not simply on whether mitochondrial biogenesis is activated, but on whether it is coordinated with mitochondrial dynamics and redox control.

### 2.2 Mitochondrial bioenergetic impairment and reduced metabolic flexibility

In obesity and T2D, insulin-target organs such as the liver, skeletal muscle, and visceral fat commonly exhibit impaired mitochondrial function, including reduced OXPHOS capacity, decreased ATP synthesis efficiency, and increased ROS production under both basal and nutrient-loaded conditions (45–47). These alterations disrupt the mitochondrial oxidation of substrates like fatty acids and glucose, creating a mismatch between nutrient influx during nutritional excess and mitochondrial oxidative capacity. Consequently, lipid intermediates accumulate in the liver and skeletal muscle, where they inhibit key insulin-signaling nodes such as IRS/PI3K/Akt. These changes further reduce metabolic resilience and promote lipotoxicity-driven insulin resistance. A cross-sectional human study reveals that offspring of T2D patients with insulin resistance but without the onset of the disease exhibit significantly reduced rates of skeletal muscle mitochondrial respiration, accompanied by intramuscular lipid accumulation and impaired glucose uptake (46). This suggests that mitochondrial dysfunction is not merely a consequence, but a key driver in the onset and progression of metabolic disorders.

Proteomic analyses comparing skeletal muscle from control individuals and T2D patients reveal that mitochondrial metabolic processes are downregulated in T2D patients (48). In pancreatic β-cells, mitochondrial uncoupling induced by metabolic stressors such as hyperglycemia and hyperlipidemia is characterized by elevated oxygen consumption, reduced ATP synthesis, and fragmented mitochondrial morphology, leading to insulin resistance and pancreatic β-cell dysfunction in diabetic conditions (49–51). Interestingly, compared with individuals without liver disease, those with non-alcoholic fatty liver disease show 30% reduction in maximal oxidative respiration of visceral adipose tissue, suggesting that adipose insulin sensitivity directly impacts mitochondrial efficiency (52). In human skeletal muscle, impact of obesity on mitochondrial proteome is subpopulation-specific: subsarcolemmal mitochondria mainly exhibit a reduction in proteins involved in protein translation, whereas intermyofibrillar mitochondria are primarily characterized by abnormalities in proteins related to energy production (53). This result suggests that obesity leads to the remodeling of mitochondrial protein composition in skeletal muscle, and this remodeling depends on the spatial location of mitochondria within the cell. Importantly, the mitochondrial pathways can re-establish energetic balance. For instance, insulin-like growth factor 2 mRNA-binding protein 2 (IMP2) deficiency boosts uncoupling protein 1 (UCP1) translation to increase thermogenesis and resist obesity. Conversely, mtDNA-encoded peptide mitochondrial open reading frame of the 12S rRNA-c (MOTS-c) activates AMPK to ameliorate insulin resistance and diet-induced weight gain. These findings highlight mitochondria can act as both drivers of metabolic dysfunction and potential therapeutic targets in obesity (54, 55). Moreover, inter-cellular mitochondrial transfer from adipocytes to macrophages, an immunometabolic “rescue” pathway, is diminished in obese adipose tissue, lowering energy expenditure and aggravating obesity (56).

### 2.3 Mitochondrial quality control

The MQC network encompasses mitochondrial division and fusion, biogenesis, protein homeostasis, and mitophagy. This system is generally impaired in the metabolic disorders obesity and diabetes. Dysfunctional MQC leads to an accumulation of damaged mitochondria, promoting oxidative stress and insulin resistance in metabolic tissues/organs (57–60). For instance, high-fat diet (HFD) fed or FUN14 domain containing 1 (FUNDC1)-deficient mice show significantly faster body weight gain, more extensive adipose tissue expansion, along with the manifestations of hyperglycemia/hyperinsulinemia/insulin resistance, compared with wild-type littermates. Furthermore, mitochondrial dysfunction in pancreatic β-cells also serves as a pivotal contributor to the pathogenesis of T2D, where impaired ATP production disrupts insulin secretion, and defective mitophagy further exacerbates pancreatic β-cell failure through the build-up of damaged mitochondria and downregulated energy metabolism genes involved in glycolysis, OXPHOS, and TCA cycle (61–63). Lipid overload in mice fed HFD impairs mitochondrial dynamics, with excess mitochondrial fission mediated by Drp1 acetylation, compromising cardiomyocyte function (64). In white adipocytes of mice, HFD activates small GTPase RalA to de-phosphorylate Drp1, promoting mitochondrial fission, lowering oxidative capacity, and accelerating weight gain. In parallel, ceramide synthase 6 (CerS6)-derived C16:0 ceramides bind mitochondrial fission factor to trigger fission, linking ectopic lipid pools to mitochondrial fragmentation and insulin resistance (65, 66). Protein homeostasis stress can also impair mitochondrial function. In obesity, mis-targeted amyloid precursor protein accumulates at the mitochondrial import gate of adipocytes and subsequently blocks protein import, diminishing mitochondrial function, and driving hypertrophy and weight gain (67). Notably, multiple animal and preclinical studies have demonstrated that pharmacological or genetic enhancement of PINK1 (PTEN induced kinase1)/Parkin-mediated mitophagy, correction of imbalance between fission and fusion, or improvement of mitochondrial protein homeostasis can alleviate tissue damage associated with obesity and diabetes. These findings suggest that MQC represents one of the key targets for intervention of metabolic diseases (29, 30, 41, 68).

### 2.4 Mitochondrial-organelle interactions, lipid and redox signaling

Mitochondrial dysfunction is not solely caused by an intrinsic mitochondrial failure, but is also greatly influenced by inter-organelle communication interfaces, particularly those with the endoplasmic reticulum (ER), as well as by the remodeling of lipid and redox signals. Mechanistically, mitochondrial Ca^2+^ handling and mitochondria-associated membrane integrity coordinate TCA/ETC (Electron Transport Chain) flux with cellular signaling, and thus their disruption contributes to insulin resistance (69). In the liver of obese mice, obesity chronically enriches mitochondria-associated ER membranes, leading to calcium overload, diminished oxidative capacity, and oxidative stress. Notably, inhibition of type I inositol 1, 4, 5-trisphosphate receptor and phosphofurin acidic cluster sorting protein 2, both of which are critical for ER-mitochondria tethering or calcium flux, can restore oxidative metabolism and improve glycemic control in obese mice (70). Furthermore, obesity blunts fasting-induced, zonated remodeling of ER–mitochondria interfaces, which are required for flexible substrate oxidation, exacerbating metabolic flexibility (70, 71). Beyond calcium handling, lipid metabolism at these contact sites couples oxidative stress to mitochondrial dysfunction. Acyl-CoA: lysocardiolipin acyltransferase 1-mediated cardiolipin re-acylation produces peroxidation-prone species, which depress respiratory chain function and promote insulin resistance (72). Additionally, the ATP binding cassette subfamily B member 10-driven export of biliverdin amplifies intracellular bilirubin redox cycling in obesity, impairing mitochondrial function and hepatic insulin action (73). Furthermore, in HFD-induced obese mice model, mtDNA leakage activates the cGAS (cyclic GMP-AMP synthase)-cGAMP (cyclic GMP-AMP)-STING (Stimulator of Interferon Genes) pathway that mediates obesity-induced metabolic dysfunction (74).

## 3 Targeting mitochondria combined with existing treatments

Although major advances have been achieved in managing obesity-related T2D, current therapeutic approaches remain limited by clinical, behavioral, and socioeconomic factors. Lifestyle modification is fundamental, but often falls short of achieving durable glycemic control and sustained weight loss due to limited long-term adherence and environmental barriers (75, 76). Nutritional interventions such as Mediterranean and low-energy ketogenic diets can produce short-term benefits, but are difficult to sustain and frequently followed by weight regain (77, 78). Pharmacologic innovations, including sodium–glucose cotransporter 2 inhibitors (SGLT2i) and glucagon-like peptide-1 receptor agonists (GLP-1RA), have expanded treatment beyond the glucose-lowering effect by offering cardiovascular and renal protection (75, 76, 79, 80). However, gastrointestinal side effects, high cost, and unequal access still restrict their global application. Similarly, dual glucose-dependent insulinotropic polypeptide (GIP)/GLP-1 agonists such as tirzepatide and once-weekly basal insulin analogues such as Icodec, efsitora further simplify therapy, but lack long-term safety and real-world outcome data (79–82). On the other hand, bariatric surgery remains the most effective intervention for sustained remission, yet invasiveness, nutritional deficiencies, and limited availability still prevent widespread implementation (37, 83). Despite meaningful reductions in morbidity and mortality, persistent challenges of affordability, tolerability, and therapeutic inertia highlight the need for more sustainable, precision-based strategies to achieve long-lasting metabolic improvement in obesity-related T2D.

In this context, mitochondrial dysfunction represents a convergent pathological node and a potential therapeutic target in obesity and T2D. This has driven the development of mitochondria-centered strategies aimed at restoring mitochondrial biogenesis, improving oxidative metabolism, reducing oxidative stress, and enhancing metabolic flexibility. Basic and translational studies have shown that activating the AMPK–PGC-1 pathway by SIRT1 activators such as resveratrol can enhance fatty acid oxidation in the liver and skeletal muscle. Thus, promoting mitochondrial biogenesis results in improved insulin sensitivity and alleviated liver steatosis (84–86). Therapeutic strategies currently under investigation include modulation of uncoupling proteins (UCPs) or ADP/ATP carrier (AAC)-mediated mild uncoupling, and mitochondrial-targeted antioxidants such as MitoQ (87, 88). At the same time, some existing drugs also partially exert their effects through mitochondria, for example, metformin has been proven to regulate ETC complex I/AMPK signal and reduce oxidative stress (89). Whereas, GLP-1 receptor agonists have been shown to improve mitochondrial membrane potential and oxygen consumption of immune cells, and downregulated inflammatory factor levels (90). In addition, exercise exerts profound regulatory effects on mitochondrial function and remains an irreplaceable strategy to improve mitochondrial health in obesity and T2D. A large number of basic and clinical studies have shown that regular exercise can significantly enhance mitochondrial biogenesis and oxidative capacity of insulin-targeted tissues such as skeletal muscle, improve metabolic flexibility, reduce lipid toxicity and chronic inflammation, thereby increasing insulin sensitivity and delaying disease progression (91–94). Collectively, among all intervention strategies exercise remains a cornerstone strategy for improving mitochondrial health. These pharmacological and lifestyle interventions position mitochondria as a central, convergent therapeutic hub, suggesting that mitochondria-focused strategies combined with existing therapies may offer a more durable metabolic relief pathway for obesity and T2D.

## 4 Advantages of *Drosophila melanogaster* as a model system for mitochondrial research

Rodent models have been widely used to study mitochondrial dysfunction in obesity and diabetes, but several limitations restrict their translational and experimental value. Physiological differences from humans, including pancreatic islet architecture and major sites of glucose disposal, may affect the interpretation of β-cell dysfunction and insulin-resistance phenotypes (95, 96). In addition, genetic background, diet composition, housing conditions, stress, and gut microbiota can strongly influence metabolic outcomes and reduce reproducibility (97–103). The high cost, long experimental timelines, and ethical constraints of rodent studies further limit large-scale genetic or pharmacological screening. These limitations support the use of complementary *in vivo* models, particularly genetically tractable systems such as *Drosophila*.

**Figure 3:**
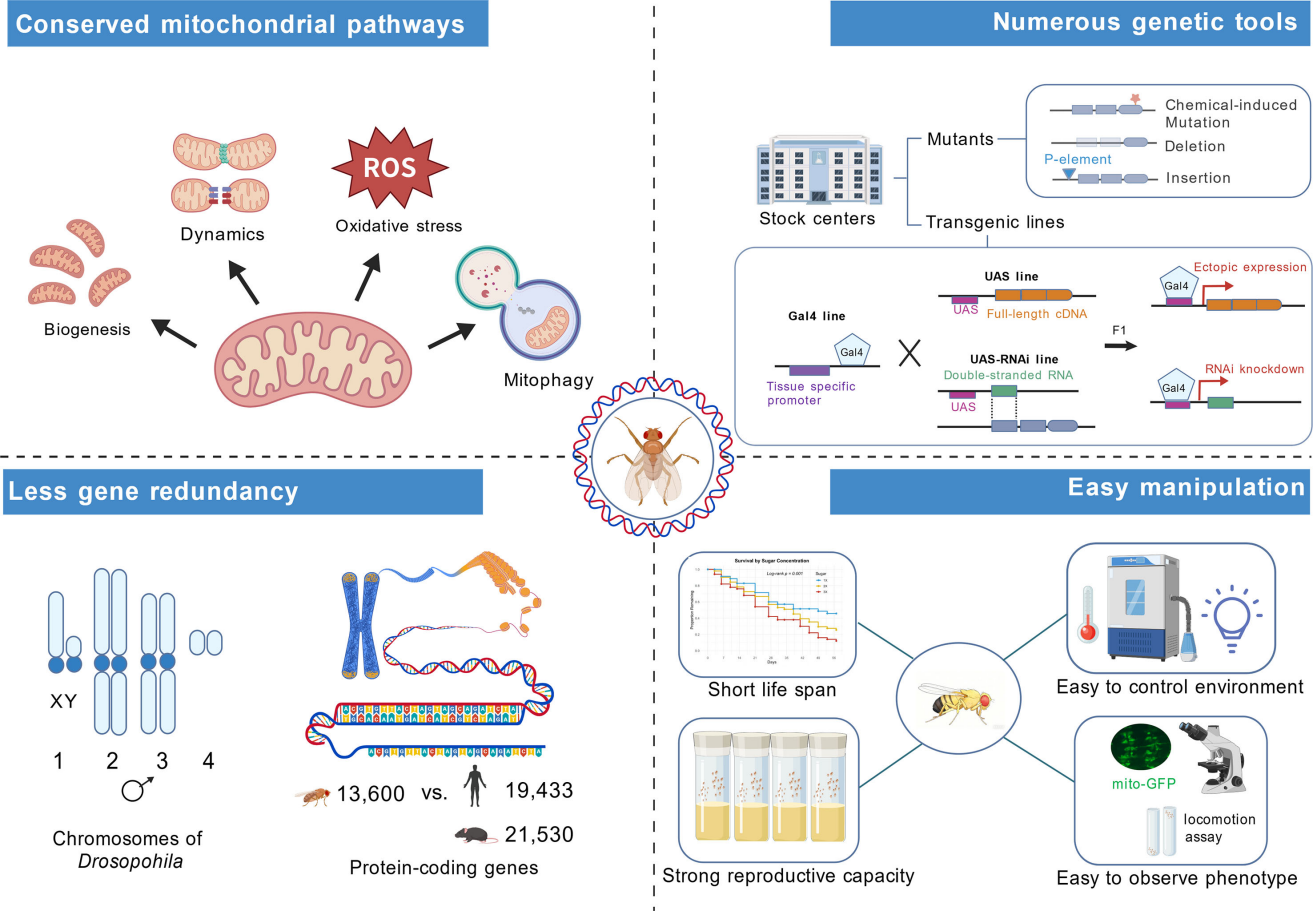
Advantages of Drosophila as a model system for mitochondrial research. Key mitochondrial pathways in Drosophila, including mitochondrial biogenesis, dynamics, oxidative stress responses, and mitophagy, are evolutionarily conserved with those in mammals, providing a foundation for studying broadly relevant mitochondrial mechanisms. Drosophila also offers extensive genetic resources, including versatile GAL4/UAS-based tools for tissue-specific cDNA overexpression or RNAi-mediated knockdown, thereby enabling precise manipulation of genes involved in mitochondrial function. In addition, Drosophila exhibits relatively low gene redundancy, with only four pairs of chromosomes and fewer protein-coding genes than mammals, helping to reduce the functional redundancy often encountered in mammalian systems. Finally, Drosophila provides major practical advantages, including a short life cycle, high reproductive capacity, ease of environmental control, and the ability to monitor mitochondrial phenotypes in vivo, collectively making it an efficient and experimentally tractable platform for studying mitochondrial biology.

Comparative genomic studies further support the use of *Drosophila* in disease modeling. An early systematic analysis of human disease-associated gene sequences identified *Drosophila* counterparts for 714 human disease genes, corresponding to approximately 77% of the disease genes examined, and the Homophila database further linked Online Mendelian Inheritance in Man (OMIM) disease genes to Drosophila gene cognates (104, 105).These findings provide a genetic basis for using *Drosophila* to model conserved disease mechanisms, including those involving mitochondrial function, insulin signaling, and metabolic regulation. Accordingly, *Drosophila melanogaster* emerges as a powerful complementary model for mitochondrial research (**Figure 3**). Despite anatomical differences, *Drosophila* exhibits highly conserved insulin/insulin-like growth factor (IGF) signaling and mitochondrial regulatory pathways, while possessing functionally analogous tissues such as insulin-producing cells, the fat body (liver- and adipose-like tissue), skeletal muscle, and oenocytes (liver-like tissue), thereby enabling whole-organism analysis of mitochondrial dysfunction under various forms of dietary stress (106–108). In addition, Drosophila’s low gene redundancy, unparalleled genetic tractability, tissue-specific GAL4/UAS tools, and CRISPR-based knock-in/knock-out approaches allow precise temporal and spatial manipulation of mitochondrial genes without the developmental compensation commonly observed in rodent knockouts (109, 110). It also supports high-throughput *in vivo* discovery through genome-wide RNAi or forward genetic screens, enabling rapid identification of polygenic modifiers of mitochondrial activity and insulin resistance that are difficult to capture in rodent models (111). Moreover, environmental and microbiota-driven variability can be tightly controlled in *Drosophila*, with defined diets, uniform housing, and tractable germ-free or gnotobiotic systems that allow the precise dissection of microbiota–mitochondria interactions far more standardized than vendor-specific microbiome variation in mice (112–115). Finally, its short life cycle, low maintenance cost, and ethical permissibility make Drosophila well suited for large-scale genetic and small-molecule screens, thereby facilitating the identification of mitochondrial pathways and candidate biomarkers that can be further evaluated in rodent models (116, 117).

Dietary *Drosophila* models have been particularly useful for recapitulating metabolic perturbations observed in human obesity and diabetes. Flies fed high-sugar diet (HSD) or HFD develop mitochondrial fragmentation in muscle tissues and expansion of adipose tissue accompanied by insulin resistance-like phenotypes, closely resembling key aspects of human metabolic dysfunction. Importantly, these dietary interventions also induce systemic abnormalities such as hyperglycemia, hyperinsulinemia, and hyperlipidemia, thereby reproducing major features of metabolic syndrome. Combined with genetic tools, these dietary paradigms enable precise dissection of glucose and lipid metabolism in *Drosophila*. For example, tissue-specific knockdown of *Drosophila* insulin-like peptides (Dilps), such as Dilp2, using the GAL4/UAS system disrupts glucose homeostasis, whereas overexpression of adipokinetic hormone (AKH), a glucagon-like factor, enhances lipid mobilization, thereby modeling diabetic hyperglycemia and ectopic lipid deposition. Together, these approaches highlight the evolutionary conservation of metabolic regulatory networks between *Drosophila* and mammals (107).

Beyond dietary manipulation, *Drosophila* also offers unique technical advantages for real-time monitoring of mitochondrial phenotypes *in vivo (*117, 118). In particular, the transparency of larval tissues enables high-resolution live imaging of mitochondrial dynamics using fluorescent markers such as mito-GFP and mito-mCherry, as well as functional reporters including mito-roGFP2-Orp1 (119–121), matrix–targeted pH-sensitive variant of GFP (mt-Keima) (122, 123), and the outer mitochondrial membrane-targeted tandem GFP–mCherry reporter mito-QC (124). Together with the GAL4-UAS system and RNAi/CRISPR libraries, these features make *Drosophila* a genetically tractable platform for interrogating conserved mitochondrial pathways *in vivo*. In addition, tens of thousands of loss-of-function mutant alleles, genome deficiency mutations, and genome-wide RNAi lines facilitate rapid generation of mitochondrial disease-related models (124). Importantly, the relatively low paralog redundancy in Drosophila further increases the likelihood that single-gene perturbations will produce detectable phenotypes (125).

The advantages of *Drosophila* also extend to the study of microbiota–mitochondria interactions. Germ-free *Drosophila* models, combined with controlled bacterial colonization, provide a streamlined system for dissecting microbiota-dependent effects on mitochondrial and metabolic homeostasis (126). For example, dietary fiber supplementation increases gut microbiota diversity and promotes mitochondrial biogenesis in the *Drosophila* brain (127). Interestingly, probiotics like *Saccharomyces boulardii* have been shown to rescue mitophagy defects in *PINK1*-null flies by restoring Parkin-dependent mitochondrial clearance, successfully linking microbial metabolites to MQC (128). Taken together, these features position *Drosophila* as a powerful platform for investigating mitochondrial biology in metabolic disorders.

Despite these strengths, *Drosophila* models also have limitations. For example, Drosophila lacks certain adipose tissue types, such as brown adipose tissue, and has a simplified physiological system. Nevertheless, humanization strategies can help bridge these gaps. By expressing human wild-type or variant proteins in Drosophila mutants, researchers can test whether the human proteins rescue *Drosophila* phenotypes and thereby assess the pathogenicity of disease-associated variants (129).

## 5 Mitochondrial dysfunction in *Drosophila* metabolic models

*Drosophila* metabolic models provide a powerful *in vivo* system for linking mitochondrial dysfunction to tissue-specific and systemic metabolic phenotypes relevant to obesity and T2D. In particular, *Drosophila* models have clarified how impaired mitophagy, altered mitochondrial dynamics, and diet-induced mitochondrial stress contribute to reduced metabolic flexibility, cardiometabolic dysfunction, diabetic nephropathy-like injury, and systemic obesity. These models therefore provide an important mechanistic bridge between conserved mitochondrial pathways and organism-level metabolic outcomes.

### 5.1 Mitophagy and mitochondrial quality control in *Drosophila* metabolic models

Although the core PINK1–Parkin pathway was first defined mainly in neurodegeneration-related models, it has also provided a conserved framework for understanding mitochondrial quality control in metabolically stressed tissues. In *Drosophila*, loss of *Pink1* causes mitochondrial dysfunction and neuromuscular degeneration, and these phenotypes can be rescued by *Parkin* overexpression, placing Parkin downstream of PINK1 (130). Subsequent studies in flies and cellular systems showed that PINK1 recruits Parkin to damaged mitochondria and promotes ubiquitination of outer mitochondrial membrane substrates such as MFN1/2, thereby coupling mitochondrial dynamics to mitophagy (131). In addition, the PINK1–Parkin pathway has been shown to support not only mitophagy but also selective turnover of respiratory-chain subunits, providing a mechanistic link between MQC failure and respiratory dysfunction (132).

Importantly, reporter systems such as mt-Keima and mito-QC allow mitophagy to be quantified directly *in vivo*. Using these tools, basal mitophagy was shown to be widespread in *Drosophila*, whereas stress-induced mitophagy is more dependent on the PINK1–Parkin pathway, indicating context-specific roles of this conserved MQC axis (124). This framework has direct relevance to metabolic disease models. In an HSD-induced diabetic nephropathy-like model, high sugar suppresses mitophagy in Malpighian tubules, leading to mitochondrial dysfunction and renal damage, whereas the mitophagy inducer PDE701 restores mitochondrial homeostasis and improves the pathological phenotype (123). Together, these studies indicate that impaired mitochondrial clearance in *Drosophila* can contribute to metabolically relevant phenotypes, particularly under nutrient stress and hyperglycemic conditions.

### 5.2 Mitochondrial dynamics and cardiometabolic dysfunction in *Drosophila* models

Mitochondrial dynamics is another major determinant of metabolic tissue function in *Drosophila*. Because abnormal mitochondrial fragmentation is closely associated with nutrient excess and organ dysfunction, *Drosophila* models are particularly useful for dissecting how altered fusion and fission contribute to metabolic pathology. Tissue-specific knockdown of *Marf* or *Opa1* causes abnormal mitochondrial morphology and a dilated cardiomyopathy-like phenotype through ROS-dependent mechanisms, while human MFN1/2 can functionally complement the *Drosophila* defects, indicating strong evolutionary conservation of mitochondrial fusion pathways (133). Diet-induced models further strengthen this link. Under HFD conditions, reduced mTORC2 activity exacerbates Drp1-mediated mitochondrial fission and cardiac dysfunction, whereas enhancement of mTORC2 signaling or inhibition of excessive fission significantly improves cardiac performance (134). These findings directly connect mitochondrial dynamics to HFD-induced cardiometabolic stress and identify the mTORC2–Drp1 axis as a conserved mechanism linking nutrient overload to cardiac mitochondrial dysfunction.

### 5.3 Diet-induced mitochondrial dysfunction and systemic metabolic imbalance

Among Drosophila studies, diet-induced and tissue-specific metabolic models provide the clearest evidence that mitochondrial dysfunction can drive obesity- and T2D-relevant phenotypes. For example, after 2 days of HFD feeding, mitochondrial respiration and ATP content in thoracic muscles increase, whereas after 4 days of HFD feeding, complex I-supported respiration is impaired and ATP content decreases (135). This is related to the increased contribution of complex II and mitochondrial glycerol-3-phosphate dehydrogenase (mG3PDH) to mitochondrial respiration. The enhanced activity of mG3PDH reflected a reduction of metabolic flexibility, leading to an imbalance and alteration of cellular redox state, ultimately resulting in cellular senescence and decreased muscle locomotor activity of *Drosophila (*135, 136). Hunter-Manseau et al. also introduced the nutritional history variable of “fasting + HFD”, showing that short-term fasting can pre-adjust mitochondrial and stress pathways to some extent, making them more resilient to subsequent HFD-induced damage (137).Tissue-specific models further show that local mitochondrial dysfunction can propagate into systemic metabolic imbalance. In particular, mitochondrial dysfunction in muscle induces systemic obesity through Activin-mediated muscle-to-fat body communication, indicating that myokine-like signaling can transmit local mitochondrial defects into remote metabolic pathology (138). Together, these studies establish Drosophila as a versatile platform for connecting mitochondrial dysfunction to reduced metabolic flexibility, cardiometabolic dysfunction, diabetic nephropathy-like injury, and systemic obesity across multiple tissues and levels of metabolic organization. Having outlined what Drosophila metabolic models have revealed about mitochondrial dysfunction, we next summarize the experimental toolkit that enables these phenotypes to be dissected *in vivo*.

Whereas Section 5 summarizes the biological insights gained from Drosophila metabolic models, the next section focuses on the experimental toolkit and quantitative readouts used to dissect mitochondrial dysfunction *in vivo*.

## 6 How to dissect mitochondrial function in metabolic disorders using *Drosophila* models

A relatively mature mitochondrial readout toolkit has been developed for Drosophila metabolic models and can be broadly categorized into mitochondrial morphology and dynamics, respiration, ROS/redox state, biogenesis and quality control, and systemic metabolic and functional readouts (**Table 1**). Rather than serving as passive markers, these readouts enable quantitative dissection of how mitochondrial dysfunction contributes to tissue-specific and systemic metabolic phenotypes *in vivo*.

**Table 1:** Mitochondrial readout toolkit in Drosophila metabolic models.

Category	Representative reporters/assays	Key readouts	Representative metabolic relevance
Mitochondrial morphology and dynamics	mito-GFP; mito-mCherry	Mitochondrial length, branching, fragmentation, network connectivity	Fusion/fission defects in metabolic stress
Respiration, ATP production, and metabolic flexibility	Seahorse XF assay; luciferase-based ATP assay	OCR, ATP content, respiratory chain activity, AMP/ATP ratio	OXPHOS defects and reduced metabolic flexibility
ROS and redox state	mito-roGFP2-Orp1; Grx1-roGFP2; HyPer; MitoTimer; DHE; MitoSOX	Mitochondrial H2O2, redox changes, oxidative stress burden	Oxidative stress and insulin resistance
Mitochondrial biogenesis and content	CS assay; mtDNA copy number; mitochondrial protein quantification	Mitochondrial abundance, respiratory capacity, biogenesis-related changes	Nutrient-dependent regulation of mitochondrial content and function
Mitophagy and MQC	mt-Keima; mito-QC; Pink1/Parkin; Atg5-related manipulations	Basal/stress-induced mitophagy; mitochondrial turnover; autophagic flux	MQC defects in HSD/HFD models
Systemic metabolic and functional readouts	tGPH; p-AKT/AKT; FoxO localization; glucose/trehalose and triglyceride assays	IIS activity; lipid homeostasis; locomotor function; lifespan; stress tolerance	Systemic metabolic dysfunction

Representative driver lines include Mef2-Gal4 (muscle), Hand-Gal4/GMH5-Gal4 (heart), and Lsp2-Gal4 (fat body).

### 6.1 Mitochondrial morphology and dynamics

Mitochondrial morphology, including volume distribution, length, branching, and fragmentation, is one of the most intuitive readouts of mitochondrial status. Usually, tissue-specific expression of mito-GFP or mito-mCherry, combined with drivers such as Mef2-Gal4 (somatic muscle), Hand-Gal4/GMH5-Gal4 (heart), and Lsp2-Gal4 (fat body), allows mitochondrial length-to-width ratio, branching, and network connectivity to be quantified by confocal microscopy. In metabolic models, these imaging approaches are particularly useful for determining whether HSD/HFD or genetic perturbations alter fusion/fission balance and mitochondrial network organization. For example, in HFD-induced cardiac models, morphology-based assays have been used to quantify Drp1-dependent mitochondrial fragmentation and to assess the impact of modulating fusion/fission pathways on mitochondrial and cardiac phenotypes (134). Thus, morphology-based imaging provides a direct framework for linking mitochondrial structural remodeling to metabolic stress in specific tissues.

### 6.2 Mitochondrial respiration, ATP production, and metabolic flexibility

At the functional level, oxygen consumption rate (OCR), ATP content, and the activity of respiratory chain complexes are key indicators for evaluating mitochondrial bioenergetics. In recent years, studies have increasingly adopted the Seahorse XF platform to determine OCR in isolated mitochondria or entire tissues, such as muscle and brain. Aw et al. established a mitochondrial isolation and respiratory testing protocol applicable to the thorax of third instar larvae and adult Drosophila, enabling the analysis of basal respiration, ATP-linked respiration, proton leak, maximal respiration, and spare respiratory capacity under different substrate and inhibitor combinations (138). In metabolic models, OCR- and ATP-based assays have been used to quantify impaired complex I-supported respiration, reduced ATP production, and reduced substrate flexibility under chronic nutrient overload (135, 136). ATP content is commonly determined using luciferase-based assays, and, when combined with the AMP/ATP ratio and AMPK phosphorylation, these measurements link mitochondrial energy state to cellular energy sensing and metabolic regulation.

### 6.3 Mitochondrial ROS and redox state

Mitochondrial ROS represents an important mechanistic bridge between metabolic stress and downstream damage signals. By measuring mitochondrial respiration together with ROS production, studies in Drosophila have examined how oxidative stress relates to mitochondrial function, lifespan, and metabolic status (139). Chemical probes such as dihydroethidium and its mitochondria-targeted derivative MitoSOX are commonly used to detect superoxide in cells and mitochondria (140). In addition, genetically encoded reporters such as mito-roGFP2-Orp1, Grx1-roGFP2, and HyPer can be expressed in specific Drosophila tissues to enable *in vivo* imaging of H_2_O_2_ dynamics and glutathione redox status in mitochondria or the cytoplasm (141, 142). The redox-sensitive fluorescent reporter MitoTimer further permits monitoring of mitochondrial oxidative stress over time and is particularly useful for mapping persistent mitochondrial damage under hyperglycemic or insulin resistance-like conditions (143). Combined with antioxidant enzyme activity assays and readouts of stress-responsive pathways, these approaches allow mitochondrial ROS load to be integrated with metabolic signaling and physiological outcomes.

### 6.4 Mitochondria biogenesis and MQC

Mitochondrial content can be evaluated through indirect indicators such as citrate synthase (CS) activity, mtDNA copy number, and mitochondrial protein abundance. A low-yeast diet was reported to reduce mitochondrial abundance and respiratory activity in adipose tissue, and “mitochondrial quality” was quantified through CS activity and mtDNA copy number (144). Through this work, Delg and Cyclin D/Cdk4 were identified as key factors linking nutrient status to the control of mitochondrial biogenesis. In addition, activation of 4E-BP enhances mitochondrial protein translation, respiratory capacity, and lifespan, further supporting the idea that mitochondrial quantity and mitochondrial activity are coordinated but distinct dimensions of metabolic regulation (145).

Mitophagy is another important readout for evaluating MQC effectiveness. Reporter systems such as mt-Keima and mito-QC allow quantitative assessment of basal and stress-induced mitophagy *in vivo*. Lee et al. used these tools to construct a basal mitophagy map in Drosophila and found that basal mitophagy is widespread across tissues (124). In metabolic models, these systems are particularly useful for determining whether HFD/HSD or IIS/TOR perturbations suppress or enhance mitophagy in tissues such as the fat body, skeletal muscle, or Malpighian tubules. For example, in HSD-induced diabetic nephropathy-like injury, mt-Keima and mito-QC have been used to detect reduced mitophagy and to evaluate pharmacological rescue of MQC defects (123, 124). Together with genetic manipulation of Atg5 and related genes, these readouts provide a quantitative framework for linking mitophagy to mitochondrial health and metabolic homeostasis.

### 6.5 Interpreting mitochondrial function within the context of systemic metabolism

Mitochondrial readouts should be interpreted together with systemic metabolic and functional phenotypes. On the one hand, IIS activity can be characterized by indicators such as the p-AKT/AKT ratio, FoxO nuclear/cytoplasmic localization, and PI3K activity measured by the fluorescent reporter tubulin-GFP-Pleckstrin Homology (tGPH). These readouts have been validated in high-glucose-induced insulin resistance models (146, 147). Standardized metabolic assays, including measurements of hemolymph glucose and trehalose, whole-body or tissue triglyceride content, and lipid droplet size and number in the fat body, enable quantification of systemic energy load and lipid homeostasis (148). Functional phenotypic readouts can also be incorporated, such as heart rate analysis by optical M-mode, locomotor performance by climbing and flight assays, and lifespan and stress-tolerance measurements (149–151). By integrating these systemic readouts with mitochondrial morphology, respiration, ROS, and mitophagy assays, mitochondrial dysfunction can be interpreted at the organ-to-system level within Drosophila metabolic models.

## 7 Conclusion

In summary, obesity and T2D represent a global metabolic crisis driven mainly by energy imbalance. Mitochondria, as the core hub connecting nutrient sensing, energy metabolism, and immune signaling, are increasingly recognized as a causal driver of metabolic flexibility loss, insulin resistance, and organ dysfunction. While existing pharmacological and lifestyle interventions can improve glycemic control and cardiovascular/renal outcomes to some extent, they often fail to fundamentally fix MQC imbalances and trans-organ energy network disorders. This suggests that mitochondrial-centered precision interventions will be a key direction for reducing the burden of obesity-related diabetes in the future. Compared with rodent models, *Drosophila* offers unique advantages in genetic manipulation, high-throughput *in vivo* screens, real-time mitochondrial imaging, and microbiota-host interaction control. It provides a powerful platform to systematically elucidate how mitochondrial biogenesis and dynamics, mitophagy, and mitochondrial-organelle interface drive metabolic disorders at the organ and systemic levels. By integrating the *Drosophila* “mitochondrial read out toolkit” with dietary and genetic interventions, and employing humanization strategies and cross-species validation, it is hoped that convertible biomarkers and therapeutic targets can be identified from the conserved mitochondrial pathways. These approaches are expected to be able to provide an experimental basis for developing mitochondrial-targeting strategies that synergize with existing hypoglycemic, weight-loss, and anti-inflammatory drugs, ultimately promoting a paradigm shift from the traditional “hypoglycemic-oriented” approach to a “mitochondrial and systemic metabolic remodeling-oriented” approach.
